# In vitro wound healing of tumor cells: inhibition of cell migration by selected cytotoxic alkaloids

**DOI:** 10.1186/s40360-018-0284-4

**Published:** 2019-01-09

**Authors:** Xiaojuan Wang, Charlotte Caroline Decker, Laura Zechner, Sonja Krstin, Michael Wink

**Affiliations:** Department of Biology, Institute of Pharmacy and Molecular Biotechnology, Heidelberg University, INF 364, D-69120 Heidelberg, Germany

**Keywords:** Cell migration, Microtubule, In vitro wound healing assay, Microtubule-binding agents, Benzophenanthridine alkaloids, Homoharringtonine

## Abstract

**Background:**

Cell migration is involved in several pathological processes such as tumor invasion, neoangiogenesis and metastasis. Microtubules are needed in directional migration.

**Methods:**

To investigate the effects of microtubule-binding agents (paclitaxel, vinblastine, colchicine, podophyllotoxin), benzophenanthridine alkaloids (sanguinarine, chelerythrine, chelidonine) and other anti-tumor drugs (homoharringtonine, doxorubicin) on cell migration, we performed the in vitro wound healing assay. The interactions between selected alkaloids and microtubules were studied via U2OS cells expressing microtubule-GFP markers.

**Results:**

The microtubule-binding natural products paclitaxel, vinblastine, colchicine and podophyllotoxin significantly altered microtubule dynamics in living cells and inhibited cell migration at concentrations below apparent cytotoxicity. The benzophenanthridine alkaloid sanguinarine, chelerythrine and chelidonine which affected microtubules in living cells, did not inhibit cell migration. Homoharringtonine (protein biosynthesis inhibitor) and doxorubicin significantly inhibited cell migration, however, they did not exert obvious effects on microtubules.

**Conclusion:**

In this study, we demonstrated that microtubule-binding agents are effective anti-migrating agents; moreover, homoharringtonine and doxorubicin can be referred as anti-migrating agents, but direct microtubule dynamics are not involved in their mode of action. Our study provides evidence that some alkaloids and other microtubule-binding natural products may be interesting candidates for the development of novel agents against metastasis.

**Electronic supplementary material:**

The online version of this article (10.1186/s40360-018-0284-4) contains supplementary material, which is available to authorized users.

## Background

Alkaloids, the largest group of secondary metabolites that produced mainly from plants, animals, bacteria and fungi, exhibit a board range of pharmacological activities such as anti-bacterial, anti-inflammatory and anti-cancer effects [1–5]. With a great diversity of structures, alkaloids interfere with various molecular targets including nucleic acids, proteins, biomembranes and neuroreceptors, among which the cytoskeleton represents one of the most important targets [6].

The ability of cells to migrate is essential for many physiological processes including embryonic development, wound repair, tumor invasion, neoangiogenesis and metastasis [7, 8]. The involvement of actin cytoskeleton in cell migration is well established [8]. In response to extracellular cues, the cell initiates the motility by setting up a front-to-back polarization, followed by a coordinated cycle of actin polymerization-dependent protrusion, integrin/actin-mediated focal adhesion and cell body translocation resulting from actomyosin contractility, which finally leads to the cell movement [9]. However, the directional migration also requires the intact microtubule cytoskeleton.

In migrating cells, an asymmetry of the microtubule network is initially established, which generates the feedbacks on Rho proteins to promote the generation of asymmetries in actin contractility and substrate adhesion, resulting in polarization and directional movement of the cell [10–13]. Microtubules fulfill different roles in cellular processes including intracellular transport, cell division and migration [14–16], making them attractive targets for natural toxins in cancer research [17, 18].

Microtubule-binding agents (MBAs) are important components in clinical combination chemotherapy and applied widely to treat many different kinds of cancers [19]. Alkaloids constitute the most important group of MBAs; well-known examples are the microtubule-stabilizer paclitaxel (a diterpene alkaloid from *Taxus* that clinically used in the treatment of Kaposi’s sarcoma, lung, ovarian and breast cancer) and the microtubule-destabilizer vinblastine (a vinca alkaloid from *Catharanthus roseus* that clinically applied for Bladder, lung and breast cancer, Hodgkin’s disease, solid tumors, leukaemia and lymphomas) [20, 21]. In the last few years, the targeting of cell migration has become a therapeutically challenging approach for cancer treatment and MBAs have also been reported to inhibit cell migration by interfering with microtubule dynamics [22].

**Fig. 1:**
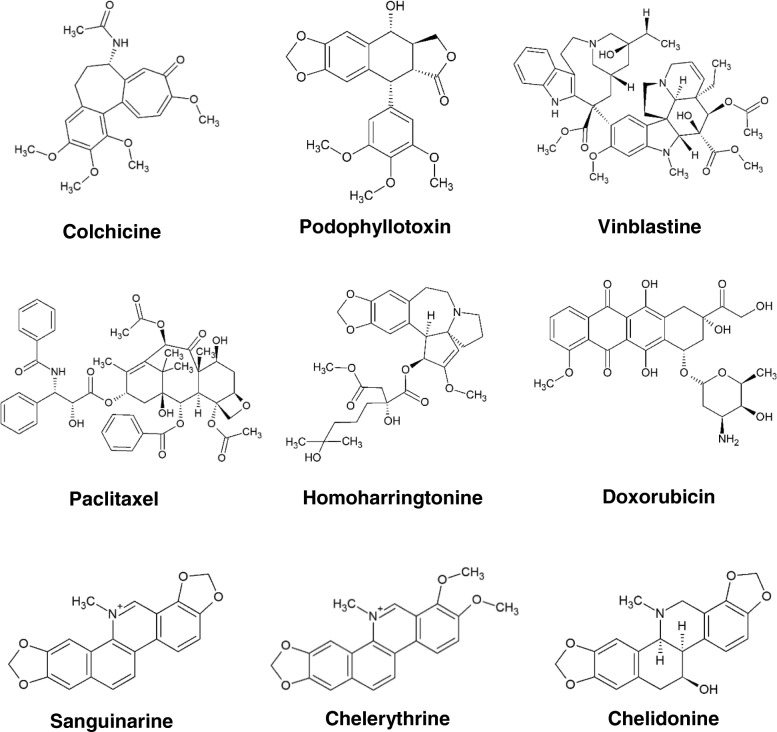
Structure of the substances tested in the study

In this study, nine cytotoxic natural products (Fig. 1) affecting different molecular targets were investigated concerning their effects on cell migration using an in vitro wound healing assay, followed by the study of their interactions with microtubules in GFP co-expressing U2OS cells. These secondary metabolites include 1) sanguinarine, a benzophenanthridine alkaloid from *Sanguinaria canadensis* that has anti-infection, anti-heart-failure, anti-inflammatory and anti-cancer effects via DNA intercalation and suppression of NF-_K_B activation [23–26]; 2) chelerythrine, a benzophenanthridine alkaloid from *Chelidonium majus* that inhibits the proliferation of neoplasms and reproduction of bacteria via DNA intercalation and inhibition of protein kinase C [27, 28]; 3) chelidonine, a benzophenanthridine alkaloid from *Chelidonium majus* that exhibits anti-inflammatory and anti-tumor activities via inhibition of telomerase and tubulin [29, 30]; 4) homoharringtonine, a cephalotaxine alkaloid from *Cephalotaxus harringtonia* that has been approved by FDA for the treatment of chronic myeloid leukemia via inhibition of protein synthesis [31, 32]; 5) doxorubicin, an anthracycline antibiotic from *Streptomyces peucetius* that has been commonly used in cancer therapy such as solid tumors, leukemia, lymphomas, breast, lung, ovarian, gastric and liver cancers for more than 40 years via inhibition of topoisomerase II [33, 34]. Microtubule-binding natural products such as paclitaxel, vinblastine, colchicine (an alkaloid from *Colchicum autumnale* that used for Familial Mediterranean fever and acute gout flares [35]) and podophyllotoxin (a lignan from *Podophyllum hexandrum* that used to treat Genital warts [36]) were investigated as positive controls. In this study we can provide evidence for partly unknown effects of these natural products on cell migration and their interactions with microtubules.

## Methods

### Chemicals

Colchicine, podophyllotoxin, dimethyl sulfoxide (DMSO), fetal bovine serum (FBS), geneticin, 3-(4,5-dimethylthiazol-2-yl)-2,5-diphenyltetrazolium bromide (MTT) were purchased from Sigma-Aldrich (Steinheim, Germany); Paclitaxel (5.95 mg/mL) and vinblastine sulfate (1 mg/mL) were obtained from the Pharmacy of the University Hospital Heidelberg (Heidelberg, Germany); sanguinarine (HPLC > 98%), chelerythrine chloride (HPLC > 98%), homoharringtonine were purchased from Baoji Herbest Bio-Tech Co., Ltd. (Baoji, Shannxi, China). Chelidonine was purchased from PhytoLab GmbH & Co. KG (Vestenbergsgreuth, Germany). Doxorubicin hydrochloride (Doxo-cell, 2 mg/mL) from cell pharm GmbH (Bad Vilbel, Germany). Dulbecco’s modified eagle’s medium (DMEM), penicillin and streptomycin from Life Technologies (Bleiswijk, Netherlands). 96-well plates and 24-well plates came from Greiner Bio-One GmbH (Frickenhausen, Germany).

### Cell culture

U2OS human osteosarcoma cancer cells, which were stably transfected with an α-tubulin-GFP construct, were supplied by Prof. Dr. Thomas Efferth (Institute of Pharmacy and Biochemistry, Johannes Gutenberg University, Mainz, Germany). U2OS-GFP-α-tubulin cells were grown in DMEM medium with 10% FBS, 1% penicillin streptomycin and continuously treated with 250 μg/mL geneticin at 37 °C and 5% CO_2_. All experiments were performed with cells in their logarithmic growth phase.

### MTT assay

The cytotoxicity of tested compounds was assessed using the MTT assay, as previously described [16]. U2OS cells (1 × 10^4^ cells/well) were seeded in 96-well plates and grown for 24 h. Then 100 μL fresh medium containing serial dilutions of compounds was added into each well and incubated for 48 h. All extracts were removed and 100 μL 0.5 mg/mL MTT solution was then added into each well. After 2 h incubation, MTT was removed and 100 μL DMSO was added. The plate was shaken at 600 rpm for 15 min and the absorbance was read at 570 nm using Tecan infinite M200 Pro (Tecan, Crailsheim, Germany). Experiments were done in triplicate, repeated three times. The IC_50_ values were calculated from concentration-response curves by SigmaPlot software (Systat Software Inc., San Jose, CA, USA). Data are presented as the mean ± standard deviation (SD).

### In vitro wound healing assay

U2OS cells (3 × 10^5^ cells/well) were seeded in 24-well plates to grow in a monolayer for 24 h. Then a sterile 20–200 μL pipette tip was held vertically to scratch a cross in each well. The detached cells were removed by washing with 500 μL PBS and shaking at 500 rpm for 5 min. 500 μL of fresh medium with or without diluted samples was added afterwards and incubated for 72 h. Before the image acquisition, the plate was washed with 500 μL pre-warmed PBS and gently shaken for 30 s. Then, pre-warmed medium or sample was added again and pictures were taken. The scratch closure was monitored and imaged in 24 h intervals using a Keyence BZ-9000 microscope (Keyence, Neu-Isenburg, Germany) at 4 x magnification and 1/3700 s exposure time.

### Analysis of open wound area

The analysis of the scratch images was performed using the TScratch Version 1.0 software [37] which calculates the scratch area (= open wound area) for each image. The percentage of open wound area was plotted over the time for each concentration. Data are presented as mean ± SD. Three to six replicates were included in the analysis and an unpaired Student’s t-test was performed. Significance was considered at *p* < 0.05.

### Fluorescence imaging

Fluorescence images of U2OS cells were taken each time after the imaging of scratch closure using the BZ-9000 microscope at 40 x magnification. For the illumination and image acquisition, the GFP channel was used and the monochromatic image was displayed in the pseudo-color green.

## Results

### Anti-proliferative activity of toxins

The anti-proliferative activity of reference drugs (vinblastine, colchicine and paclitaxel) and cytotoxic alkaloids (sanguinarine, chelerythrine, chelidonine and homoharringtonine) in U2OS cells has been previously studied by us [22]. In this study, we included data on the cytotoxicity of doxorubicin and podophyllotoxin (Table 1). The known anti-tumor drug doxorubicin inhibited the growth of U2OS cells with an IC_50_ value of 0.69 μM. The microtubule-binding natural products colchicine, vinblastine, podophyllotoxin and paclitaxel showed more potent anti-proliferative activities than doxorubicin with IC_50_ values between 0.1 nM and 0.23 μM. Vinblastine exhibited the strongest inhibition with an IC_50_ value of 0.10 nM, whereas homoharringtonine caused the second strongest cytotoxicity with an IC_50_ value of 3 nM. The benzophenanthridine alkaloids sanguinarine, chelerythrine and chelidonine are also cytotoxic; they inhibited the growth of U2OS cells with IC_50_ values ranging between 0.92 μM and 3.86 μM.

**Table 1:** The cytotoxicity of selected natural products in U2OS cells

Compounds	IC_50_
Vinblastine	0.10 ± 0.05 nM
Homoharringtonine	3.00 ± 1.67 nM
Colchicine	10.67 ± 10.18 nM
Podophyllotoxin	33.6 ± 4.31 nM
Paclitaxel	0.23 ± 0.10 μM
Doxorubicin	0.69 ± 0.26 μM
Sanguinarine	0.92 ± 0.53 μM
Chelerythrine	2.88 ± 0.76 μM
Chelidonine	3.86 ± 1.99 μM

Data are presented as the mean ± standard deviation (SD).

### Do selected alkaloids interfere with cell motility in vitro?

**Fig. 2:**
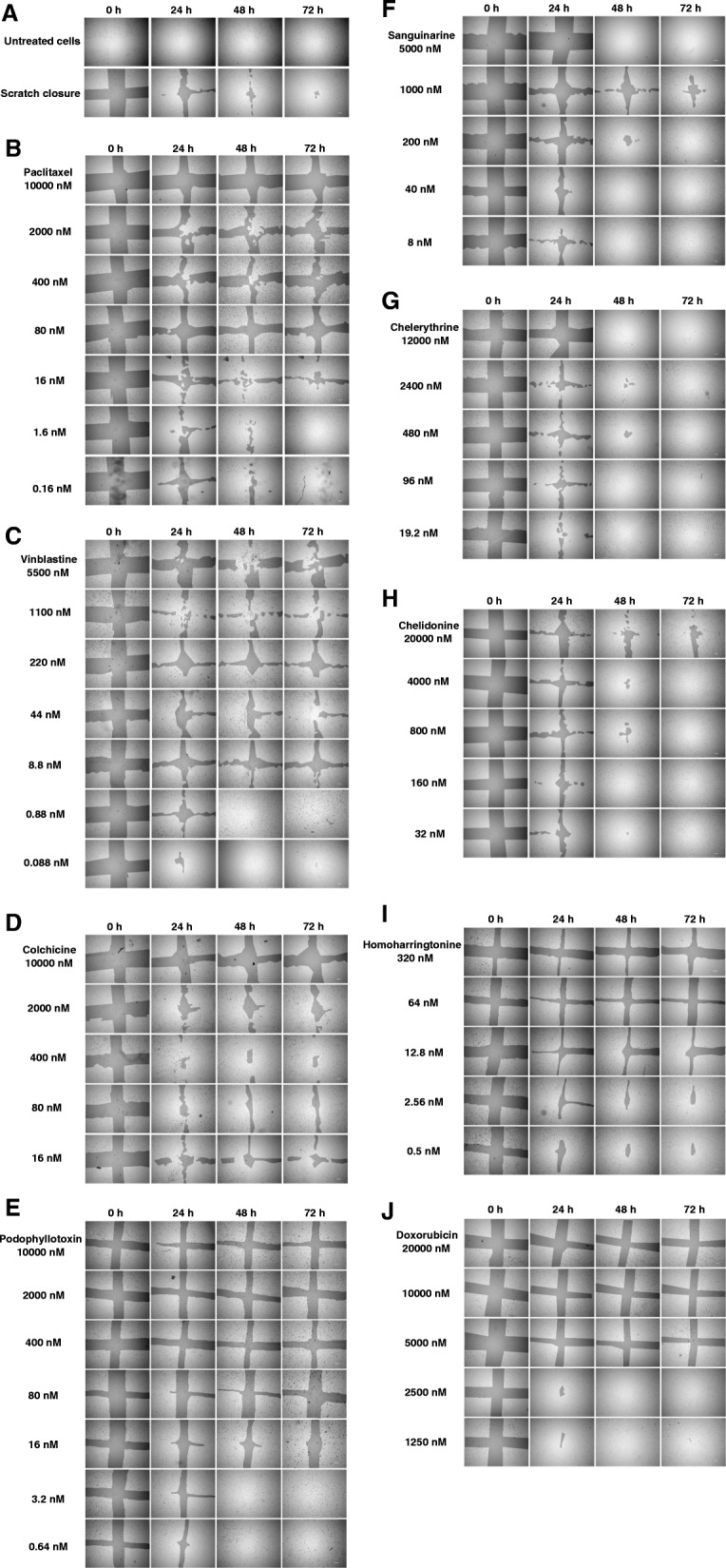
Time course of scratch closures with tested drugs**.** (**a**) Time course of scratch closure in the absence of drugs. The four upper images show untreated cells, while the four lower images illustrate the size of the scratch area (analysis with TScratch software). (**b** - **j**) U2OS monolayers were mechanically wounded with a 20–200 μL sterile pipette tip following treatment with tested alkaloids. Bar = 200 μm

In the wound healing assay, we examined cell migration in response to the mechanical scratch wound in the absence or presence of putative inhibitors. Images of scratch areas from the time points 0, 24, 48 and 72 h are illustrated in Fig. 2. Figure 2a shows the representative control at each time point indicating that the scratch was half closed within 24 h and completely closed after 72 h.

**Fig. 3:**
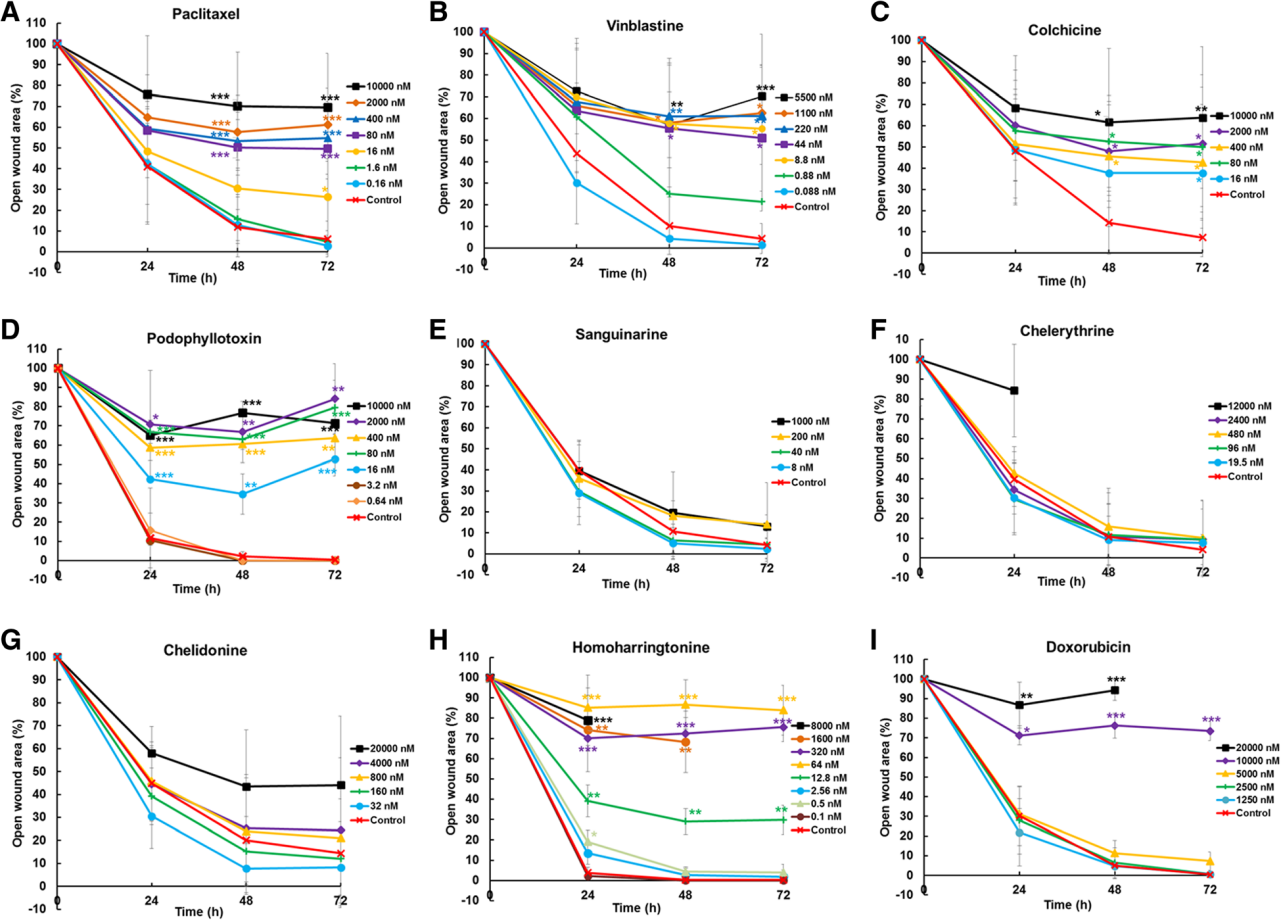
The relative size of open scratch area. (**a** - **i**) The open wound area was measured after the treatment of tested alkaloids. The scratch area of untreated cells is set to 100%. * *p* < 0.05, ** *p* < 0.01, *** *p* < 0.001

To quantify the effects of putative migration inhibitors, the percentage of the open wound area after 72 h was determined (Table 2 and Fig. 3). Our data clearly shows that treatment with homoharringtonine, doxorubicin and microtubule-binding agents paclitaxel, vinblastine, colchicine and podophyllotoxin caused a significant inhibition of cell migration in a concentration-dependent manner.

**Table 2:** Open scratch area of U2OS cells treated with putative migration inhibitors. The scratch area at time 0 was set 100%; *** *p* < 0.001, ** *p* < 0.01, * *p* < 0.05; n = number of repetitions. / = cells detached from flask surface

Compound	Concentration (nM)	24 h		48 h		72 h		n
Paclitaxel	Control	41.03 ± 27.83		11.91 ± 14.40		6.09 ± 8.54		6
	10,000	75.67 ± 28.21		70.15 ± 25.93	***	69.49 ± 25.96	***	6
	2000	64.68 ± 20.47		57.75 ± 17.49	***	61.11 ± 17.56	***	6
	400	59.24 ± 16.44		53.24 ± 14.03	***	54.84 ± 17.46	***	6
	80	58.47 ± 13.84		50.23 ± 13.39	***	49.62 ± 14.53	***	6
	16	48.24 ± 12.56		30.49 ± 18.99		26.34 ± 18.47	*	6
	1.6	42.11 ± 27.84		15.68 ± 11.85		4.79 ± 5.37		4
	0.16	42.37 ± 19.51		12.86 ± 7.46		2.89 ± 4.23		3
Vinblastine	Control	43.80 ± 32.59		10.22 ± 13.33		4.35 ± 6.95		4
	5500	72.66 ± 19.76		57.06 ± 15.12	**	70.21 ± 14.25	***	4
	1100	65.92 ± 31.03		58.11 ± 29.74	*	62.65 ± 36.28	*	4
	220	67.60 ± 27.12		60.92 ± 23.69	**	61.14 ± 22.50	**	4
	44	63.52 ± 28.10		55.48 ± 30.19	*	50.97 ± 33.90	*	4
	8.8	69.83 ± 23.52		57.55 ± 30.94	*	55.31 ± 32.09	*	4
	0.88	60.74		25.08		21.46		2
	0.088	30.21		4.30		1.44		2
Colchicine	Control	48.05 ± 24.45		14.30 ± 14.71		7.31 ± 8.94		5
	10,000	68.25 ± 24.54		61.62 ± 34.50	*	63.70 ± 33.17	**	5
	2000	60.03 ± 25.98		47.87 ± 23.46	*	51.51 ± 32.26	*	5
	400	51.41 ± 25.58		45.61 ± 24.09	*	42.70 ± 27.24	*	5
	80	57.52 ± 23.55		52.54 ± 21.60	*	49.97 ± 27.97	*	5
	16	48.62 ± 25.82		37.68 ± 25.16		37.71 ± 26.11	*	5
Compound	Concentration (nM)	24 h		48 h		72 h		n
Podophyllo- toxin	Control	11.66 ± 3.82		2.22 ± 2.36		0.43 ± 0.75		3
	10,000	65.10 ± 4.24	***	76.78 ± 1.37	***	71.37 ± 5.90	***	3
	2000	70.81 ± 28.15	*	66.87 ± 15.93	**	84.08 ± 18.24	**	3
	400	58.66 ± 6.67	***	60.56 ± 2.13	***	63.58 ± 12.85	**	3
	80	66.88 ± 6.17	***	62.97 ± 5.34	***	79.59 ± 14.17	***	3
	16	42.41 ± 1.51	***	34.57 ± 10.57	**	52.73 ± 8.81	***	3
	3.2	10.45 ± 14.19		0		0		3
	0.64	15.70 ± 22.11		0		0		3
Sanguinarine	Control	39.69 ± 13.67		10.73 ± 10.21		4.19 ± 7.67		4
	5000	/		/		/		5
	1000	39.62 ± 12.17		19.70 ± 5.54		13.10 ± 5.47		5
	200	35.96 ± 18.07		18.12 ± 20.85		13.96 ± 20.04		5
	40	29.66 ± 7.64		6.43 ± 7.75		4.37 ± 6.48		5
	8	28.96 ± 15.04		5.07 ± 7.42		2.39 ± 4.84		5
Chelerythrine	Control	39.69 ± 13.67		10.73 ± 10.21		4.19 ± 7.67		4
	12,000	84.35 ± 23.35	*	/		/		5
	2400	34.43 ± 12.35		10.83 ± 14.73		9.34 ± 16.31		5
	480	42.79 ± 13.29		15.89 ± 19.22		10.02 ± 18.69		5
	96	29.69 ± 18.00		11.70 ± 21.10		9.28 ± 19.95		5
	19.5	30.33 ± 17.73		9.07 ± 18.47		7.64 ± 16.94		5
Chelidonine	Control	44.80 ± 16.45		20.16 ± 22.85		14.38 ± 23.73		5
	20,000	57.98 ± 11.72		43.41 ± 24.82		44.06 ± 30.05		5
	4000	44.59 ± 15.78		25.26 ± 23.38		24.46 ± 31.62		5
	800	45.80 ± 17.05		23.94 ± 23.20		21.03 ± 24.67		5
	160	39.21 ± 12.32		15.26 ± 15.19		12.04 ± 16.10		5
	32	30.50 ± 14.01		7.77 ± 11.29		8.22 ± 16.72		5
Homoharring- tonine	Control	3.76 ± 2.83		0.31 ± 0.69		0.26 ± 0.58		3
	8000	78.98 ± 12.39	***	/		/		3
	1600	74.23 ± 20.66	**	68.25 ± 15.17	**	/		3
	320	70.09 ± 6.42	***	72.37 ± 7.95	***	75.60 ± 7.17	***	3
	64	85.25 ± 16.02	***	86.73 ± 12.13	***	83.94 ± 12.26	***	3
	12.8	39.11 ± 7.84	**	28.99 ± 6.44	**	29.76 ± 7.19	**	3
	2.56	13.45 ± 5.53		2.68 ± 3.97		1.95 ± 3.38		3
	0.5	19.08 ± 5.65	*	4.31 ± 1.71		3.95 ± 3.51		3
	0.1	2.18 ± 0.85		0		0		3
Doxorubicin	Control	30.29 ± 15.09		5.06 ± 4.76		0.36 ± 0.62		3
	20,000	86.77 ± 11.70	**	94.33 ± 5.34	***	/		3
	10,000	71.22 ± 4.93	*	76.25 ± 6.40	***	73.47 ± 4.82	***	3
	5000	31.19 ± 2.67		11.32 ± 6.42		7.27 ± 4.59		3
	2500	27.93 ± 17.02		6.43 ± 7.73		0.65 ± 1.12		3
	1250	21.82 ± 17.07		4.76 ± 6.55		0.58 ± 1.01		3

Data are presented as the mean ± SD.

Between concentrations of 10 μM and 80 nM, paclitaxel significantly inhibited the scratch closure after 48 h and deformed the cell morphology. The effect of 16 nM paclitaxel was less obvious, while no migration inhibition were observed below 16 nM. Compared with paclitaxel, vinblastine inhibited cell migration even stronger: it significantly inhibited the scratch closure at a low concentration of 8.8 nM, which also changed the cell shape from irregular into round form within 24 h. No influence on cell motility was observed after treatment with 0.88 nM and 0.088 nM vinblastine. The migration of cells was impaired by colchicine at all applied concentrations, ranging from 10 μM to 16 nM. Similar to colchicine, 10 μM to 16 nM podophyllotoxin also significantly inhibited cell migration.

The benzophenanthridine alkaloids did not inhibit cell motility: Even 5 μM sanguinarine did not disturb cell migration but it detached the cells from the bottom of the culture flask within 24 h, making it impossible to determine the scratch size. No effect on cell motility and cell morphology was recorded for sanguinarine concentrations below 5 μM (Fig. 3e and Table 2).

Similar to sanguinarine, chelerythrine detached cells at the highest applied concentration (12 μM) after 24 h of incubation, which made it impossible to measure the scratch area. Lower concentrations of chelerythrine (< 12 μM) did not affect cell migration and morphology significantly (Fig. 3f and Table 2).

Figure 3g indicates that 20 μM chelidonine might inhibit the scratch closure, however, this effect was not statistically significant (Table 2). At this concentration, chelidonine also detached cells and induced a change in cell morphology. Below the concentration of 2 μM, chelidonine did not differ from the untreated control.

Similar to microtubule-binding agents, homoharringtonine inhibited the wound healing at low concentrations; cells were completely detached from the flask surface at a concentration of 8 μM after 48 h and 1.6 μM after 72 h. However, the scratch closure was significantly inhibited at the concentration of 12.8 nM (Table 2).

Treatment of the cells with 20 and 10 μM doxorubicin caused inhibition of cell motility and change of cell morphology within 24 h. Although the scratch usually closed after the treatment with 5 μM doxorubicin, the cell morphology showed changes after 48 h.

### Do selected alkaloids interfere with microtubules in living cells?

**Fig. 4:**
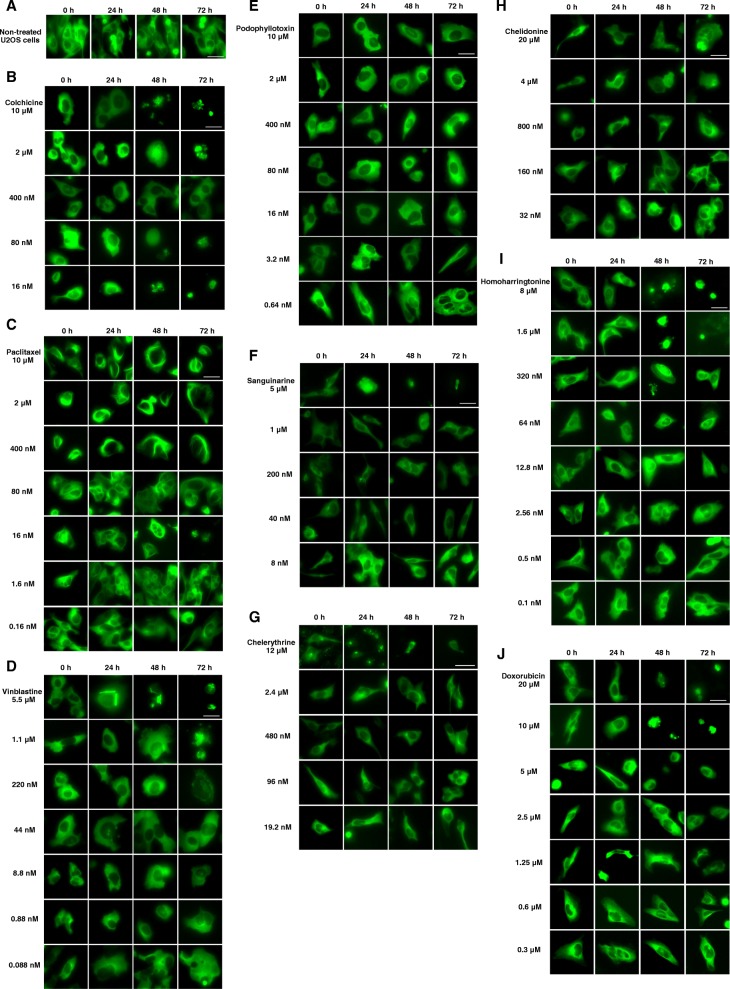
Representative fluorescence images of U2OS cells treated with tested substances. In U2OS cells microtubules carry a GFP label which allowed to visualize the microtubule network. Panels show micrographs of U2OS cells treated for 0 h, 24 h, 48 h and 72 h without (**a**) or with (**b** - **j**) tested alkaloids at different concentrations. Bar = 30 μm

In order to determine whether the inhibition of cell migration caused by selected toxins was mediated by an alteration of microtubule dynamics, U2OS cells were imaged under fluorescence microscopy. Figure 4 illustrates the dose dependence of each compound on the microtubule network. In non-treated U2OS cells, the microtubules extended continuously through the cytoplasm and formed an extensive intracellular network with the exception of the nuclear region (Fig. 4a).

Microtubule-binding agents which significantly inhibited cell motility in previous experiment, also affected microtubules in living U2OS cells. The microtubule-stabilizer paclitaxel promoted the polymerization of microtubules with the brightness and thickness increased over time (Fig. 4c). The microtubule morphology was changed after the treatment with 1.6 nM paclitaxel, which had no effect on cell migration.

Treatment with microtubule-destabilizers induced microtubule depolymerization. 8.8 nM and 5.5 μM vinblastine extensively reduced the mass and depolymerized the network of microtubules (Fig. 4d). These concentrations also inhibited the cell motility. At a concentration of 5.5 μM, tubulin paracrystals were formed and dispersed through the cytoplasm. Cells treated with vinblastine were seen to lose their cellular protrusions and changed their morphology. This effect was even visible at low concentrations (0.88 nM and 0.088 nM) that did not affect cell migration, with the appearance of multi-nucleated cells.

Except for the formation of tubulin paracrystals, colchicine showed similar effects on microtubules as vinblastine. Colchicine mainly reduced the mass of the microtubule network which appeared less dense at the cell periphery compared to the cells at the start of the experiment (Fig. 4b). Podophyllotoxin exhibited a similar effect as colchicine; the mass of microtubule network was decreased and the cell morphology was changed (Fig. 4e).

Though the benzophenanthridine alkaloids did not significantly inhibit cell migration, they influenced the microtubule network to some extent. Sanguinarine appeared to reduce the mass of microtubule network and to change the cell morphology with concentrations increased (Fig. 4f). Compared to other substances, 12 μM chelerythrine exerted a stronger effect on microtubules in that the microtubule network was apparently disrupted and depolymerized within minutes (Fig. 4g 0h), which finally led to apoptosis. At lower concentrations, chelerythrine exhibited similar effects on microtubules as sanguinarine. Similar to colchicine and podophyllotoxin, chelidonine mainly decreased microtubule mass at the cell periphery at all applied concentrations (Fig. 4h).

The treatment of homoharringtonine (Fig. 4i) significantly changed cell morphology and reduced microtubule mass at the concentrations (12.8 nM – 8 μM) that also interfered with cell motility. Below 12.8 nM, the cell morphology and microtubule mass were slightly altered by homoharringtonine.

Doxorubicin (Fig. 4j) exhibited strong effects both on cell morphology and reduction of microtubule mass at high applied concentrations (1.25–20 μM). Below concentrations of IC50 (0.3–0.6 μM), no obvious changes were observed on microtubules and cell morphology.

## Discussion

The present study elucidated the effects of nine cytotoxic natural products on cell migration and their putative interactions with microtubules. Doxorubicin, homoharrigtonine and microtubule-binding agents paclitaxel, vinblastine, colchicine and podophyllotoxin significantly inhibited cell migration, but not the benzophenanthridine alkaloid sanguinarine, chelerythrine and chelidonine. In addition, all substances exhibited certain effects on microtubules.

Paclitaxel, vinblastine, colchicine and podophyllotoxin are known as MBAs that stabilize or destabilize microtubules. Our previous study [38] had demonstrated that paclitaxel, vinblastine and colchicine alter microtubule dynamic both in living cells and in vitro. In present study, the interaction between these substances and cellular microtubules was further investigated (Fig. 4b-e), which is in accordance with our previous findings. Here we further provide evidence that podophyllotoxin inhibited tubulin polymerization in vitro with IC_50_ value of 2.04 μM (Additional file 1: Table S1 and Additional file 2: Figure S1), indicating its mode of action consists with colchicine, vinblastine and paclitaxel. In addition, these MBAs also inhibited cell migration at the concentrations which were lower than those causing cytotoxicity. Paclitaxel has been reported to inhibit cell migration at concentrations that significantly suppress microtubule dynamics without modifying the microtubule mass, which limited the number of microtubule plus ends to regulate the formation of lamellipodia. Belotti D et al. [39] also demonstrated that the anti-migratory effect of paclitaxel occurs at non anti-proliferative concentrations in tumor cells. Vinblastine was also found to suppress both dynamic instability and cell migration at low concentration, but higher drug concentrations are needed to inhibit microtubule assembly and cell division [22]. Our observations are in accordance with these published data, which indicates the potential ability of MBAs to act as effective anti-migrating agents. MBAs inhibit cell motility by interference with microtubule dynamics, preventing the activation of Rac1/Cdc42 and disorganizing the actin cytoskeleton [40].

Consistent with recent studies [38, 41], benzophenanthridine alkaloid sanguinarine, chelerythrine and chelidonine were found to affect microtubule network and change the cell morphology in U2OS cells (Fig. 4f-h), further demonstrating that an interaction occurs between benzophenanthridine alkaloids and microtubules. We have previously reported that sanguinarine and chelerythrine which inhibited microtubule polymerization in vitro, did not cause mitotic arrest [38]. In the present study, we found that sanguinarine, and chelerythrine did not inhibit the migration of U2OS cells like other microtubule-binding agents (Table 2), which agrees with the previous assumption that the mode of sanguinarine and chelerythrine differs from that of microtubule-binding agents. Furthermore, the microtubule dynamics are probably not their main cause to induce apoptosis. Chelidonine, which altered microtubule dynamics and induced mitotic arrest in the previous study [38], did not significantly inhibit cell migration. In contrast to our findings, several authors have reported the inhibition of migration by benzophenanthridine alkaloids. JP Eun and GY Koh [42] found that sanguinarine inhibited cell migration in endothelial cells by blocking VEGF-induced Akt activation; I Tan et al. [43] have noticed that chelerythrine inhibited the speed of migration in U2OS cells by blocking cellular activity of MRCK; O Kim et al. [44] reported that chelidonine suppressed migration and invasion of MDA-MB-231 cells by inhibiting formation of the integrin-linked kinase/PINCH/α-parvin complex. So how can we explain this discrepancy? Different types of cells contain specific factors which may modulate their sensitivities to these alkaloids. In addition, sanguinarine, chelerythrine and chelidonine affect multiple targets such as DNA (intercalation), protein kinase C (inhibitor) and telomerase (inhibitor) etc. The specific factors of cancer cells and the multiple targets of these alkaloids therefore result in various responses in different cell types, which requires further systematic research to understand the anticancer properties of this class of compounds and the pathways that lead to apoptosis.

Homoharringtonine is a known protein synthesis inhibitor that has been used clinically to treat chronic myeloid leukemia [45, 46]. Homoharringtonine displayed the second strongest cytotoxicity among the tested compounds during the study. In addition, homoharringtonine significantly inhibited cell migration in the wound healing assay, which has been reported for the first time. At high applied concentrations, homoharringtonine exhibited strong effects on cell morphology and microtubule mass. However, disruption of tubulin polymerization and cell cycle are not involved in the mechanisms of homoharringtonine [38]. Thus, a probable explanation might be that the inhibition of protein biosynthesis would also prevent the synthesis of proteins related to the cytoskeleton.

Doxorubicin is an anthracycline antibiotic that has been commonly used in the cancer treatment for more than 40 years [34, 47]. Doxorubicin is known to induce various severe side effects in clinical application including poor wound healing [48, 49]. In this study, we observed that doxorubicin inhibited cell migration, altered cell morphology, and reduced microtubule mass at high cytotoxic concentrations in U2OS cells, which could explain these clinical reports. However, doxorubicin did not show impact on tubulin assembly in vitro (Additional file 1: Table S1), indicating that direct effects on microtubules are not involved in its mechanism. Doxorubicin impaired cell migration probably via the inhibition of protein synthesis and prolyl 4-hydroxylase [49]. Generally, doxorubicin could be referred as an anti-migrating agent but its clinical administration should be further studied and more research is needed to improve the therapeutic efficiency and decrease its side effects.

## Conclusions

This study investigated the roles of cytotoxic alkaloids in biological processes related to cell migration and cytoskeleton dynamics. In conclusion, we found that: (1) MBAs are effective anti-migrating agents; (2) the benzophenanthridine alkaloid sanguinarine, chelerythrine and chelidonine did not inhibit cell migration; (3) homoharringtonine and doxorubicin can be referred as anti-migrating agents, but direct microtubule dynamics are not involved in their mode of action. Our study emphasize the link between microtubule inhibitors and inhibition of cell migration. More studies at a molecular level are necessary to understand the exact role of microtubule inhibitors in cell invasions and metastasis.

## Additional files

Additional file 1: **Table S1.** The IC_50_ values of podophyllotoxin and doxorubicin on tubulin polymerization. Each experiment was independently performed three times. (DOCX 14 kb)

Additional file 2: **Figure S1.** Podophyllotoxin inhibited tubulin polymerization in vitro. In-Vitro tubulin polymerization assay was performed according to a standard protocol (Reference 16). Polymerization of tubulin with MAPs in the assembly buffer was measured in the absence (◆) and in the presence of different concentrations of podophyllotoxin. Podophyllotoxin significantly inhibited the nucleation and growth phase during microtubule assembly. (DOCX 59 kb)

## Abbreviations

DMSO: Dimethyl sulfoxide
FBS: Fetal bovine serum
MBAs: Microtubule-binding agents
MTT: 3-(4,5-dimethylthiazol-2-yl)-2,5-diphenyltetrazolium bromide
