# Supplementary material for: In vitro wound healing of tumor cells: inhibition of cell migration by selected cytotoxic alkaloids
Source: BMC Pharmacol Toxicol. 2019 Jan 9;20:4. doi: 10.1186/s40360-018-0284-4 (PMC6327619; doi:10.1186/s40360-018-0284-4)
Supplement: Supplementary file 1 — Table S1. The IC50 values of podophyllotoxin and doxorubicin on tubulin polymerization. Each experiment was independently performed three times. (DOCX 14 kb) [file 40360_2018_284_MOESM1_ESM.docx]

**Table 1**. The IC_50_ values of podophyllotoxin and doxorubicin on tubulin polymerization. Each experiment was independently performed three times.

| **Compound** | **IC_50_** |
| --- | --- |
| Podophyllotoxin | 2.04 ± 0.16 µM |
| Doxorubicin | > 1 mM |

Data are shown as mean ± SD
