# Supplementary material for: In vitro wound healing of tumor cells: inhibition of cell migration by selected cytotoxic alkaloids
Source: BMC Pharmacol Toxicol. 2019 Jan 9;20:4. doi: 10.1186/s40360-018-0284-4 (PMC6327619; doi:10.1186/s40360-018-0284-4)
Supplement: Supplementary file 2 — Figure S1. Podophyllotoxin inhibited tubulin polymerization in vitro. In-Vitro tubulin polymerization assay was performed according to a standard protocol (Reference 16). Polymerization of tubulin with MAPs in the assembly buffer was measured in the absence (◆) and in the presence of different concentrations of podophyllotoxin. Podophyllotoxin significantly inhibited the nucleation and growth phase during microtubule assembly. (DOCX 59 kb) [file 40360_2018_284_MOESM2_ESM.docx]

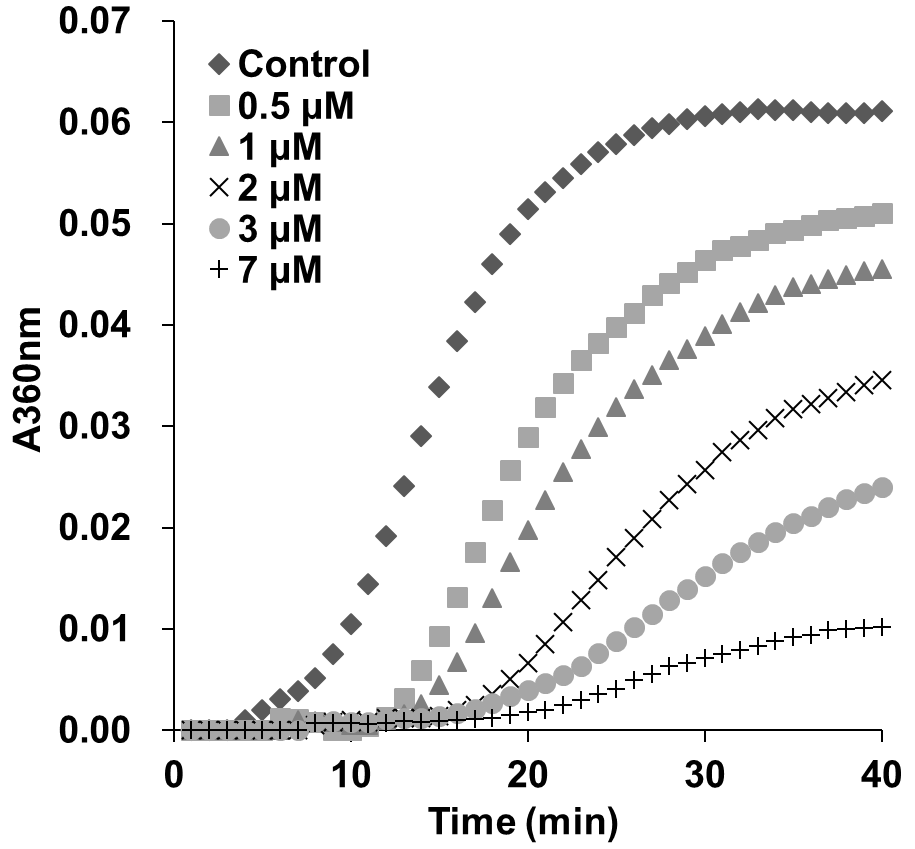


**Figure 1. Podophyllotoxin inhibited tubulin polymerization *in vitro***. *In-Vitro* tubulin polymerization assay was performed according to a standard protocol (Reference 16). Polymerization of tubulin with MAPs in the assembly buffer was measured in the absence (◆) and in the presence of different concentrations of podophyllotoxin. Podophyllotoxin significantly inhibited the nucleation and growth phase during microtubule assembly.
